# PRIC295, a Nuclear Receptor Coactivator, Identified from PPAR*α*-Interacting Cofactor Complex

**DOI:** 10.1155/2010/173907

**Published:** 2010-09-05

**Authors:** Sean R. Pyper, Navin Viswakarma, Yuzhi Jia, Yi-Jun Zhu, Joseph D. Fondell, Janardan K. Reddy

**Affiliations:** ^1^Department of Pathology, Feinberg School of Medicine, Northwestern University, Chicago, IL 60611, USA; ^2^Department of Physiology and Biophysics, Robert Wood Johnson Medical School, UMDNJ, Piscataway, NJ 08854, USA

## Abstract

The peroxisome proliferator-activated receptor-*α* (PPAR*α*) plays a key role in lipid metabolism and energy combustion. Chronic activation of PPAR*α* in rodents leads to the development of hepatocellular carcinomas. The ability of PPAR*α* to induce expression of its target genes depends on Mediator, an evolutionarily conserved complex of cofactors and, in particular, the subunit 1 (Med1) of this complex. Here, we report the identification and characterization of PPAR*α*-interacting cofactor (PRIC)-295 (PRIC295), a novel coactivator protein, and show that it interacts with the Med1 and Med24 subunits of the Mediator complex. PRIC295 contains 10 LXXLL signature motifs that facilitate nuclear receptor binding and interacts with PPAR*α* and five other members of the nuclear receptor superfamily in a ligand-dependent manner. PRIC295 enhances the transactivation function of PPAR*α*, PPAR*γ*, and ER*α*. These data demonstrate that PRIC295 interacts with nuclear receptors such as PPAR*α* and functions as a transcription coactivator under *in vitro* conditions and may play an important role in mediating the effects *in vivo* as a member of the PRIC complex with Med1 and Med24.

## 1. Introduction

Lipid metabolism in mammals is a complex process regulated by diverse factors including the members of the nuclear receptor subfamily known as peroxisome proliferator-activated receptors (PPARs). There are three isoforms of PPAR known as PPAR*α*, PPAR*β*/*δ*, and PPAR*γ* [1–3]. PPAR*α*, the initial isoform identified, is centrally involved in the pleiotropic responses induced in rat and mouse liver by treatment with peroxisome proliferators [4–7]. Peroxisome proliferators are structurally diverse chemicals and include compounds such as clofibrate, bezafibrate, nafenopin, and others along with phthalate ester plasticizers, certain herbicides, pesticides, industrial solvents, and leukotriene D_4_ receptor antagonists [8, 9]. The pleiotropic responses induced following treatment with a peroxisome proliferator include the lowering of serum lipids, the proliferation of hepatic peroxisomes, and the coordinated induction of fatty acid oxidation genes involved in the mitochondrial and peroxisomal *β*-oxidation, and microsomal *ω*-oxidation pathways [10]. Additionally, rats and mice chronically exposed to peroxisome proliferators develop a high incidence of hepatocellular carcinomas even though these compounds are nongenotoxic and considered a paradigm for epigenetic carcinogenesis [5, 6, 11]. PPAR*α* also exerts a significant role in the process of inflammation [12–15]. All these responses, including the development of hepatocellular carcinomas, are receptor mediated and achieved through selective activation of PPAR*α* [16–18]. 

PPARs heterodimerize with retinoid X receptor-*α* (RXR*α*) and bind to peroxisome proliferator response elements (PPREs) present in the promoter region of target genes [19–22]. In the absence of cognate ligand, transcription of target genes is repressed by corepressor proteins such as silencing mediator of retinoic acid and thyroid hormone receptors (SMRT) [23], nuclear receptor corepressor (NCoR) [24, 25], and receptor interacting protein 140 (RIP140) [26] bound to the PPAR-RXR heterodimers. Upon ligand binding, PPARs undergo a conformational change causing the dissociation of corepressor proteins and then allowing for the orchestrated recruitment of coactivator proteins [27, 28]. These coactivator proteins enhance transcription through various means including histone acetyltransferase activity, methyltransferase activity or mediating the interaction of the activated PPAR-RXR heterodimer with the basal transcription machinery of the cell [29–35].

Coactivators and coactivator-associated proteins known to be involved in PPAR*α*-mediated transactivation include three members of the SRC (steroid receptor coactivator)/p160 family of proteins [29, 32, 36], CBP (CREB-binding protein)/p300 [35, 37, 38], PBP (PPAR*γ*-binding protein)/MED1/TRAP220/DRIP205 [30, 39–45], PRIP/NCoA6/ASC2/RAP250/TRBP/NRC [46–50], PIMT/NCoA6IP [31], CARM1[51], PRIC285 [33], PRIC320/CHD9/CReMM [34, 52], PGC-1*α* [53], PGC-1*β*, and others [54, 55]. Each of these coactivator proteins contains one or more conserved LXXLL (L = leucine, X = any  amino  acid) motifs which are necessary for the interaction with the activation function-2 (AF-2) domain present at the C-terminal end of a nuclear receptor [56]. Although several cofactors have been shown to interact with PPARs and function as transcriptional regulators under *in vitro* conditions, there is limited data on the *in vivo* functions of these molecules in PPAR-regulated target gene transcription. To date, deletion of Med1 in liver has been shown to abrogate the ability of PPAR*α* to activate transcription of target genes as well as block other pleiotropic effects of receptor activation including liver regeneration and the development of hepatocellular carcinoma [57]. Furthermore, Med1 deletion also affects PPAR*γ*-regulated adipogenesis in mouse embryonic fibroblasts and the adipogenic steatosis induced by PPAR*γ* overexpression in liver [58, 59]. Med1 was first cloned using PPAR*γ* as bait in the yeast two-hybrid system and subsequently detected in the PPAR*α*-interacting cofactor (PRIC) complex of approximately 25 proteins isolated from rat liver nuclear extracts [30, 33]. The identities of proteins in the PRIC complex [33] revealed several coactivators previously identified in yeast two-hybrid screens such as Med1 [30], CBP [35], SRC-1 [29], Med24/TRAP100 [60], PIMT [31], and PGC-1 [53]. The PRIC complex also included some novel proteins including PRIC285 and others [33]. Subsequently, this approach was modified to use ciprofibrate, a synthetic PPAR*α* agonist, to pull down a complex of proteins from rat nuclear extract and this resulted in the identification of PRIC320/CHD9 and some other high molecular weight proteins [34]. In this study, we present data to show that one of the hitherto uncharacterized high molecular weight proteins, designated PRIC295, contains 10 LXXLL coactivator motifs and interacts with PPAR*α* in a ligand-dependent manner. PRIC295 significantly enhances the transcriptional activity of PPAR*α* *in vitro*. We further demonstrate that PRIC295 interacts with the Med1 and Med24 subunits of the Mediator complex suggesting that PRIC295 may be important for nuclear receptor signaling *in vivo*.

## 2. Materials and Methods

### 2.1. Preparation of Liver Nuclear Extracts and Ciprofibrate-Sepharose Beads for Affinity Pulldown

Liver tissue was harvested from 5 male F-344 rats sacrificed 1 hour after an intragastric dose of ciprofibrate (250 mg/kg body weight). Nuclei were isolated and extract prepared as described elsewhere [33, 34]. Ciprofibrate was immobilized on AH-Sepharose 4B by carbodiimide reaction as previously described [61]. Animals were housed under a 12 hour light/12 hour dark cycle and maintained in individual cages with standard rodent chow and water *ad libitum. *All animal procedures employed in this study were reviewed and approved by the Institutional Animal Care and Use Committee (IACUC) at Northwestern University. 

### 2.2. Affinity Pulldown and Matrix-Assisted Laser Desorption/Ionization-Time of Flight

Ciprofibrate-sepharose beads were blocked with 1% fatty-acid free bovine serum albumin and washed with buffer containing 0.5% NaCl. Nuclear extract (∼10 mg protein) was allowed to interact with Sepharose beads with immobilized ciprofibrate overnight at 4°C. After washing, bound proteins were eluted from the beads by boiling and resolved by SDS-PAGE on a 3%–8% Tris-acetate gel or 4%–20% acrylamide gel as described previously [34]. Gels were stained with silver nitrate to visualize the resolved proteins and selected bands were cut and processed for matrix-assisted laser desorption/ionization-time of flight (MALDI-TOF) analysis using a Voyager DE Pro at the IMSERC Laboratory at Northwestern University. Observed peaks were identified by the use of MS-FIT (Protein Prospector, University of California, San Francisco). To isolate PRIC295 interacting proteins, nuclear extracts were allowed to interact with GST-ΔPRIC295^840–1815^ (F2) protein and the bound proteins were subjected to MALDI-TOF analysis.

### 2.3. PRIC295 Cloning and Plasmid Constructs

The human full-length cDNA (KIAA0219) was purchased from the Kazusa DNA Research Institute, Chiba, Japan. Full-length expression vectors were made by restriction digestion of the cDNA and ligation into the pcDNA3.1(+) expression vector (Invitrogen). Smaller fragments of the cDNA were amplified using the high fidelity rTth polymerase (Applied Biosystems) and ligated into pcDNA3.1(+), pGEX-5X-1 (GE Healthcare), or pM (Clontech) vectors at the designated sites to give the plasmids pcDNA-ΔPRIC295^1–915^ (Fragment1—F1), pcDNA-ΔPRIC295^840–1815^ (F2), and pcDNA-ΔPRIC295^1740–2671^ (F3). These fragments were also ligated into pGEX-5X-1 and pM. All final vectors were confirmed by sequencing at the Genomics Core Facility at Northwestern University. *In vitro* translated proteins were made using the TnT T7 quick-coupled transcription/translation kit from Promega according to the manufacturer's protocol and radiolabeled with L-^35^S-methionine (Perkin Elmer). Other plasmids including pGEX-PPAR*α*, pCMX-PPAR*α*, pGEX-PPAR*γ*, pCMX-PPAR*γ*, pGEX-ER*α*, pcDNA-ER*α*, pGEX-CAR, pGEX-TR*α*, pGEX-RXR*α*, pCMX-RXR*α*, CMV-RL, pGL3-3xPPRE-LUC, PGL3-3xERE-LUC, GST-Med1, and cloned fragments of Med1 fused to GST have all been previously described [30, 33, 62].

### 2.4. Northern Blotting and Quantitative Real-Time PCR

Northern blotting for human PRIC295 mRNA expression was performed using a multiple tissue blot purchased from Clontech and the 3′-terminal 1 kb of the PRIC295 cDNA as a probe. Total RNA for quantitative PCR was isolated using TRIzol reagent (Invitrogen) according to the manufacturer's instructions. Quantitative PCR was done using an ABI 7300 (Applied Biosystems). Samples were tested in triplicate and normalized with 18S ribosomal RNA. Specific PCR products were measured by melting curve analysis and relative gene expression changes were measured using the comparative C_T_ method, X = 2^−ΔΔCT^. PRIC295 qPCR primers were designed from exons 27 and 28.

### 2.5. GST-Pulldown with PRIC295 and Nuclear Receptors

GST and GST-fusion proteins were purified using glutathione sepharose 4B beads (GE Healthcare) and incubated with *in vitro* synthesized PRIC295 labeled with ^35^S-methionine (or fragments of PRIC295). Binding was allowed to take place on a gentle shaker at 4°C for 2 hours, then bound protein was washed, eluted, and resolved by SDS-PAGE and analyzed by autoradiography as described earlier [30, 46]. Quantification of binding was performed using ImageJ software available from the NIH.

### 2.6. Transactivation Assays

The ability of PRIC295 to enhance transcriptional activation mediated by PPAR*α*, PPAR*γ*, or ER*α* was measured by transfecting HeLa cells with appropriate plasmids using Lipofectamine 2000 (Invitrogen) according to the manufacturer's protocol. Plasmids used in these assays were pCMX-PPAR*α*, pCMX-PPAR*γ*, pcDNA-ER*α*, pCMX-RXR*α*, pGL3-3xPPRE-Luc, pGL3-3xERE-Luc, and pcDNA3.1-PRIC295. Experiments were conducted in triplicate and luciferase expression was assessed using the dual-luciferase reporter assay system from Promega according to the manufacturer's protocol. Ligands used in these experiments (Wy-14,643 for PPAR*α*, rosiglitazone for PPAR*γ*, and 17-*β*-estradiol for ER*α*) were added at a concentration of 100 *μ*M. Transcriptional activity of PRIC295 was further examined by transfecting HeLa cells with pM-ΔPRIC295^1–915^ (F1), pM-ΔPRIC295^840–1815^ (F2), or pM-ΔPRIC295^1740–2671^ (F3) which express chimeric proteins containing fused fragments of PRIC295 with the DNA-binding domain (aa 1–147) of the yeast Gal4 transcription factor [63]. These cells were also transfected with the Gal4-TK-Luc plasmid containing the c-terminal portion of the Gal4 protein and the thymidine kinase promoter upstream of the luciferase reporter gene. The ability of chimeric Gal4-PRIC295 proteins to activate transcription of the luciferase reporter gene was analyzed by comparison to cells transfected with Gal4-TK-luc and empty pM vector [64].

### 2.7. Immunofluorescence, Immunoblotting, and Coimmunoprecipitation

HeLa cancer cells were transfected with pCMX-PPAR*α* and pcDNA-PRIC295-3xFLAG using lipofectamine 2000 reagent. Cells were fixed 48 hours posttransfection with 4% paraformaldehyde for 20 minutes and stained with anti-PPAR*α* (Santa Cruz Biotechnology Inc.—sc-9000) and anti-FLAG monoclonal antibody M2 (Sigma). Fluorescence microscopy and digital image photographs were obtained using a Nikon Eclipse E600 microscope equipped with a Spot RT slider digital camera and image analysis software (Diagnostic Instruments). Immunoblotting was performed to ascertain the presence of PPAR*α*, Med1, or Med24/TRAP100 in the PRIC complex of proteins isolated using ciprofibrate-Sepharose and proteins that bind PRIC295 in the GST-pulldown assay. For coimmunoprecipitation, HeLa cells were transfected with pcDNA3.1-PRIC295-3xFLAG and pCMX-PPAR*α* expression vectors as previously described. Nuclear extract was made and interacting proteins were purified using resin covalently bound with the anti-FLAG M2 antibody (Sigma). Proteins were subjected to SDS-PAGE, then immunoblotted using anti-FLAG, anti-Med1 (Santa Cruz Biotechnology, Inc.—sc-5334), or anti-PPAR*α*.

### 2.8. Statistical Analysis

Statistical significance of the difference between transfected groups of cells used in the luciferase assays were calculated by one-tailed Student's *t*-test using the available functions in Microsoft Excel 2007.

## 3. Results and Discussion

### 3.1. Identification of PRIC295

**Figure 1:**
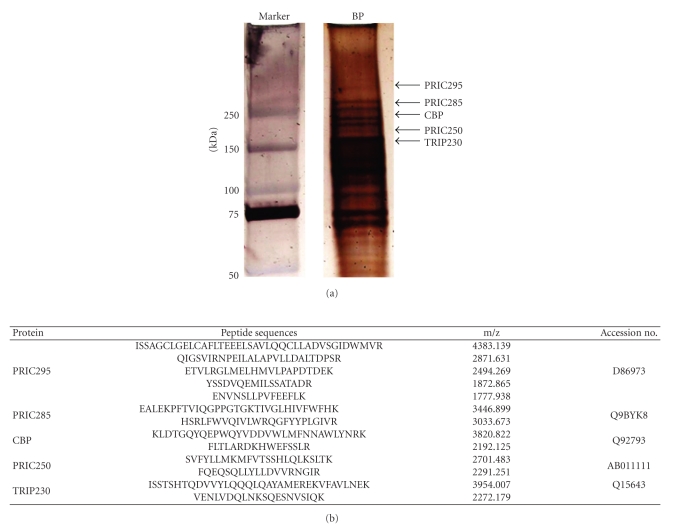
PRIC295 in ciprofibrate bound protein complex. (a) Ciprofibrate-binding protein complex isolated from rat liver nuclear extracts analyzed on a 3%–8% Tris-acetate silver stained gel. Lane 1 marker; lane 2 BP, binding proteins. Selected high molecular weight bands (arrows) were excised from the gel and digested with trypsin. MALDI-TOF analysis was conducted on the marked bands yielding peptide-matching protein identities, in descending order from highest molecular weight to lowest, of PRIC295, PRIC285, CBP, PRIC250, and TRIP230. (b) Mass spectrometric and limited sequence analysis data of proteins identified by MALDI-TOF with their associated peptide fragment sequences, m/z ratios and corresponding accession numbers.

To identify rat liver proteins that interact with peroxisome proliferators we initially used an affinity chromatography approach that involved ciprofibrate immobilized on AH-Sepharose 4B by carbodiimide reaction [61]. This procedure resulted in the isolation of peroxisome proliferator-binding proteins that ranged in apparent Mr of 31,000–79,000 [61], but they remained uncharacterized due to the limited availability and applicability at that time of MALDI-TOF technology [65]. The general availability of MALDI-TOF mass spectrographic technology in recent years enabled the analysis of liver nuclear proteins that bind to GST-fused full-length PPAR*α* [33], or to AH-Sepharose-ciprofibrate affinity matrix [34]. In the present study, we describe the identification of a member of the PRIC complex from ciprofibrate-bound proteins (Figure 1(a)) which has been designated as PRIC295 based on molecular mass. Mass spectrographic analysis of one of the high molecular weight protein bands revealed several peptide fragments that matched the human KIAA0219 protein (accession number D86973, reference sequence NM_006836) (Figure 1(b)). Human PRIC295 shares 94% homology with the rat and 93% homology with the mouse orthologues. Other proteins identified by MALDI-TOF in the PRIC complex include PRIC285, CREB-binding protein (helicase CBP), PRIC250, and thyroid receptor-interacting protein 230 (TRIP230) (Figure 1(b)). In this complex, we also identified previously known PPAR*α* cofactor proteins that include PRIC285 and PRIC320 (data not shown).

### 3.2. Molecular Characterization of PRIC295

**Figure 2:**
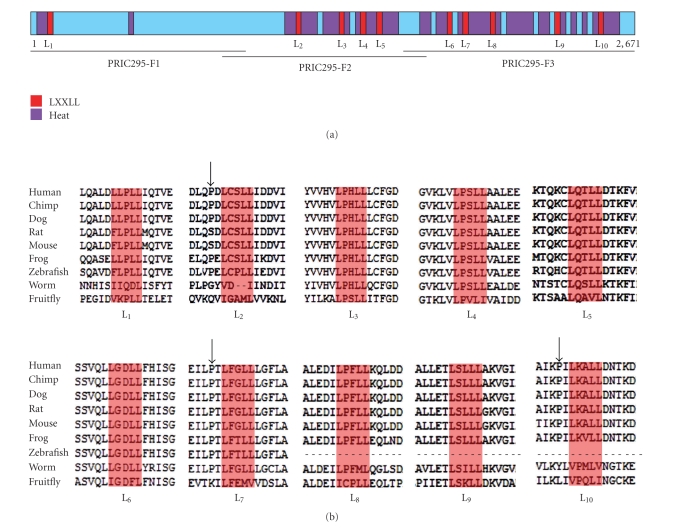
PRIC295 structural features. (a) Schematic representation of the PRIC295 protein to indicate the locations of LXXLL motifs in red color and HEAT repeat domains in purple color. (b) Alignment of the orthologous PRIC295 genomic sequences of selected species at the loci of all 10 of the LXXLL motifs contained in the human PRIC295 protein. LXXLLs are numbered L_1_ to L_10_ beginning from the N-terminal region: L_1_aa465–469; L_2_aa1160–1164; L_3_aa 1461–1465; L_4_aa1499–1503; L_5_aa1596–1600; L_6_aa1839–1843; L_7_aa1926–1930; L_8_aa2045–2049; L_9_aa2330–2334; L_10_aa2594–2598. The model organism to which each sequence corresponds is indicated at the left side of the sequence. The location of conserved proline at—2 of L_2_, L_7_, and L_10_ are indicated using an arrow.

The gene encoding PRIC295 is located on human chromosome 12q23.24 and consists of 58 exons. The nucleotide sequence data for PRIC295 are available in the Third Party Annotation Section of the DDBJ/EMBL/GenBank databases under the accession number TPA: BK006575. The 8,666 bp long transcript has an open reading frame of 8013 bp that encodes a 2,671 aa protein of approximately 295 kDa (accession number NP_006827.1). PRIC295 contains 10 LXXLL coactivator motifs located at aa L_1_,465–469; L_2_,1160–1164; L_3_,1461–1465; L_4_,1499–1503; L_5_,1596–1600; L_6_,1839–1843; L_7_,1926–1930; L_8_,2045–2049; L_9_,2330–2334; L_10_,2594–2598 (Figure 2(a)). The LXXLL motifs are significant in coactivator and activator (receptor) interactions. In PRIC295 these motifs are evolutionarily conserved across many different species (Figure 2(b)). Sequences flanking these conserved LXXLL motifs have also been shown to be influential in determining the specificity of the interaction between coactivators and activators. Three of the LXXLL motifs, namely, L_2_, L_7_, and L_10_, present in PRIC295 possess a conserved proline in the -2 position relative to the first leucine of the LXXLL motif (Figure 2(b)). The presence of proline at this position is similar to the biologically important LXXLL motifs known to interact with PPAR*α* such as those found in Med1 [30].

In addition to LXXLL motifs, the PRIC295 sequence also reveals the presence of 24 HEAT repeats (see Table 1 in Supplementary Material available online at doi:10.1155/2010/173907) which may play a role in protein-protein interactions as well as energy production and conversion (NCBI—Conserved Domain Database—COG1413) (Figure 2(a)) [66]. HEAT repeat domains are composed of approximately 50 hydrophobic and charged amino acids conserved at particular positions within the motif [67]. These motifs are believed to be important in mediating protein-protein interactions though the exact mechanism by which this occurs is not clear. The presence of HEAT repeats in PRIC295 places it in a class of HEAT repeat proteins known to be involved in gene transcription and translation, but also have other functions [67]. It is possible that the HEAT repeats in PRIC295 may be important in mediating interactions between different transcription cofactors within the PRIC complex.

### 3.3. Tissue Distribution of PRIC295 Transcripts

**Figure 3:**
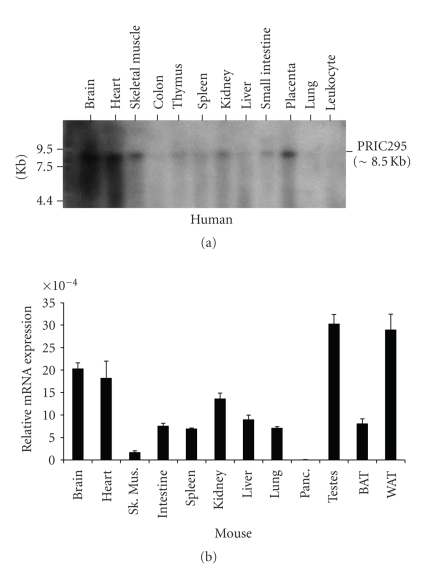
Expression of PRIC295 in human and mouse tissues. (a) Northern blot analysis of PRIC295 mRNA expression using a human multiple tissue blot (Clontech). Blot contains 2 *μ*g of polyA RNA in each lane from tissues indicated. PRIC295 transcript (∼8.5 kb) is expressed in nearly all tissues. (b) Quantitative PCR data showing the expression of PRIC295 mRNA in several different mouse tissues. Primers were designed to amplify from the exon 27-28 region of the cDNA sequence. Samples were normalized with 18S RNA. RNA from 5 different C57B6/J males was pooled and samples were run in triplicate. Standard deviations are shown.

Northern blot analysis revealed that the PRIC295 mRNA transcript is 8.5 kb in length and is expressed in many different human tissues with the highest expression observed in brain, heart, skeletal muscle, and placenta (Figure 3(a)). In thymus, spleen, liver, and small intestine PRIC295 expression was evident whereas in lung and colon the transcript was barely detectable. The expression of PRIC295 in different mouse tissues was evaluated by qPCR and the data show that it is expressed robustly in testes, white adipose tissue, brain, heart, and kidney (Figure 3(b)). Embryonic expression of PRIC295 mRNA in the mouse was also evaluated using the qPCR approach and the data suggest higher levels of expression during developmental stages E15.5 and E16.5. By E18.5 the expression level was low (Supplementary Figure 1). *In situ* hybridization data for PRIC295 mRNA localization during various developmental stages in the mouse should provide clues about the expression in tissues at different developmental stages. 

### 3.4. Interaction of PRIC295 with Nuclear Receptors

**Figure 4:**
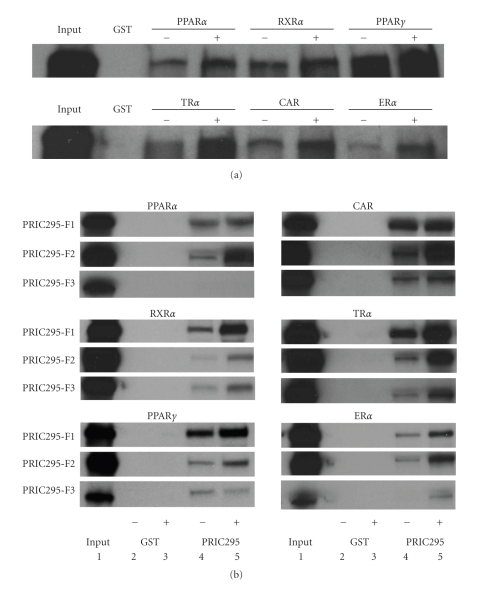
PRIC295 interactions with nuclear receptors. (a) *In vitro* interaction of radiolabeled full-length PRIC295 with nuclear receptors PPAR*α*, RXR*α*, PPAR*γ*, TR*α*, CAR, and ER*α*. PRIC295 was radiolabeled using ^35^S-methionine (Perkin Elmer) and incubated with each shown receptor fusion protein in GST-pulldown assay. (−) minus indicates *in vitro* binding interaction in the absence of cognate ligand and (+) plus indicates the presence of receptor-specific ligand during the *in vitro* binding interaction. Ligands used: Wy-14,643 for PPAR*α*; 9-cis-retinoic acid for RXR*α*; rosiglitazone for PPAR*γ*; triiodothyronine (T3)TR*α*; 1,4-Bis[2-(3,5-dichloropyridyloxy)]benzene (TCPOBOP) for CAR; 17-*β*-estradiol for ER*α*. All ligands were used at a concentration of 100 *μ*M. (b) Interaction of radiolabeled PRIC295 fragments ΔPRIC295^1–915^ (F1), ΔPRIC295^840–1815^ (F2), and ΔPRIC295^1740–2671^ (F3) with GST-fusion nuclear receptor proteins. Loading for each lane is indicated at the base of each panel. Lane 1 input; lane 2 GST alone without ligand; lane 3 GST alone with receptor-specific ligand; lane 4 GST-receptor fusion in the absence ligand; lane 5 in the presence of receptor-specific ligand. All ligands used are as previously described in (a).

Interactions between PRIC295 and PPAR*α* and some other members of the nuclear receptor superfamily were analyzed by GST-pulldown assays. These were performed using bacterially-expressed GST-fusion nuclear receptor proteins and *in vitro* translated pcDNA-PRIC295 (Figure 4(a)). Full-length PRIC295 bound *in vitro *to PPAR*α*, PPAR*γ*, RXR*α*, CAR, ER*α*, and TR*α* and addition of receptor specific ligand (10 *μ*M to 100 *μ*M) enhanced this binding (Figure 4(a); Supplementary Figure 2). Ligands used for each receptor were pirinixic acid (Wy-14,643) for PPAR*α*, rosiglitazone for PPAR*γ*, 9-cis-retinoic acid for RXR*α*, TCPOBOP for CAR, 17-*β*-estradiol for ER*α*, and triiodothyronine (T3) for TR*α*. PRIC295 did not bind to GST alone, as expected (Figure 4(a)).

To further investigate which region of PRIC295 protein might be involved in receptor interactions, additional GST-pulldown assays were undertaken using the GST-fusion receptor proteins and *in vitro* translated PRIC295 fragments designated ΔPRIC295^1–915^ (F1), ΔPRIC295^840–1815^ (F2), and ΔPRIC295^1740–2671^ (F3) (Figure 4(b)). Fragment F1 contains 1 LXXLL (L_1_), fragment 2 contains 4 LXXLLs (L_2_–L_5_), and fragment 3 contains 5 LXXLLs (L_6_–L_10_) (Figure 2(a)). The fragment ΔPRIC295^1–915^ (F1) with 1 LXXLL (L_1_) bound strongly to GST-PPAR*γ*, GST-RXR*α*, and GST-TR*α*, while ΔPRIC295^840–1815^ (F2) bound strongly to GST-PPAR*α*, GST-CAR, and GST-ER*α*. In general, F1 and F2 bound more avidly than F3 to these receptors. Although PRIC295 fragments F1 and F2 bound to PPAR*α*, surprisingly, there was no perceptible interaction of this receptor with fragment ΔPRIC295^1740–2671^ (F3) although F3 contains 5 LXXLLs (Figures 2(a) and 4(b)). The relative binding levels were digitally quantified (Supplementary Figure 3). These observations suggest that PRIC295 might exert a role in mediating the transcription of target genes of several members of the nuclear receptor superfamily. Since different nuclear receptors exhibited different affinities for interacting with the 3 different fragments of PRIC295, it is possible that different LXXLL motifs may determine the binding to a given nuclear receptor. In this regard, fragment 1 (F1) which has only one LXXLL (L_1_) may be important biologically as this fragment binds more readily than the other two fragments. Additional studies are needed to modify this first LXXLL and selected others for binding specificity.

### 3.5. PRIC295 Enhances PPAR*α*, PPAR*γ*, and ER*α*-Mediated Transcription in Mammalian Cells

**Figure 5:**
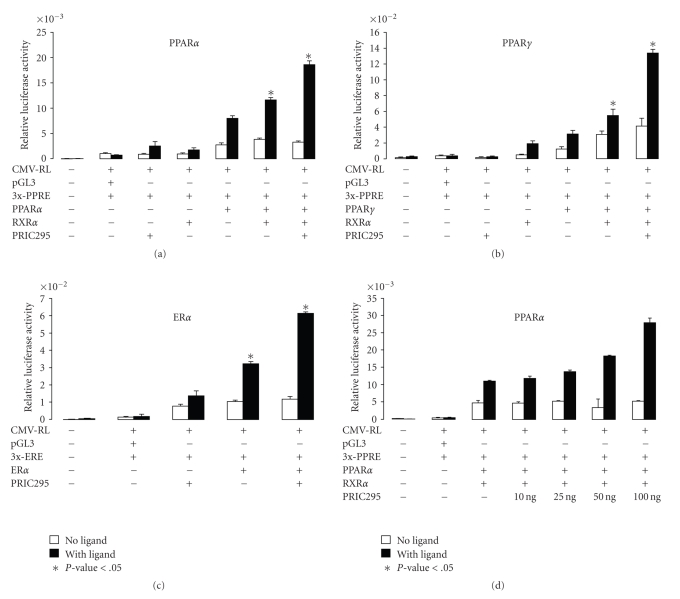
PRIC295 functions as a transcriptional coactivator for nuclear receptors. Data are shown for PPAR*α* (a), PPAR*γ* (b), and ER*α* (c). pGL3-3xPPRE-LUC was used as a reporter for PPAR*α* and PPAR*γ* whereas 3xERE was used as reporter for ER*α*. HeLa cells were transfected with 100 ng of pcDNA-PRIC295, pCMX-PPAR*α*, pCMX-pCMX-PPAR*γ*, pCMX-ER*α*, pCMX-RXR*α*, pGL3-3xPPRE-LUC (or empty pGL3-LUC vector), and 50 ng of CMV-RL plasmid as indicated (+ or −). Each column shown is the mean relative luciferase unit value for triplicate experiments. Open bar represents activity in the absence and dark bar in the presence of respective ligand (see Figure 4). All experiments were normalized against the expression of *Renilla* luciferase (CMV-RL) as an internal control. Statistical significance (*P*-value) is indicated at the base of the panel and calculated by comparing transfected groups marked with an *. (d) Transcriptional activation of PPAR*α* with the addition of the increasing amounts of PRIC295. All other plasmids were transfected using the same amounts indicated above.

In order to examine the functional relevance of the interaction between PRIC295 and nuclear receptor proteins, we transiently coexpressed PRIC295 with pCMX-PPAR*α*/pCMX-RXR*α* (Figure 5(a)), with pCMX-PPAR*γ*/pCMX-RXR*α* (Figure 5(b)), or with pcDNA-ER*α* (Figure 5(c)) in HeLa cells. These cells were concurrently transfected with luciferase reporters pGL3-3xPPRE-LUC for PPAR*α* and PPAR*γ*, or pGL3-3xERE-LUC for ER*α* in order to measure the ability of PRIC295 to transactivate receptor-mediated transcription. PRIC295 clearly increased the transcriptional activation of the luciferase reporter gene in a ligand-dependent manner when cotransfected with pCMX-PPAR*α*, pCMX-PPAR*γ*, or pcDNA-ER*α* (Figures 5(a)–5(c)). PRIC295 also enhanced PPAR*α*/RXR*α*-mediated transcriptional activation of pGL3-3xPPRE-LUC in a dose-dependent manner (Figure 5(d)). PRIC295 also enhanced PPAR*α*/RXR*α*-mediated transcription in the presence of the RXR*α* ligand 9-cis-retinoic acid (Supplementary Figure 4). These results confirm that PRIC295 functions as a coactivator for nuclear receptors PPAR*α*, PPAR*γ*, and ER*α*.

**Figure 6:**
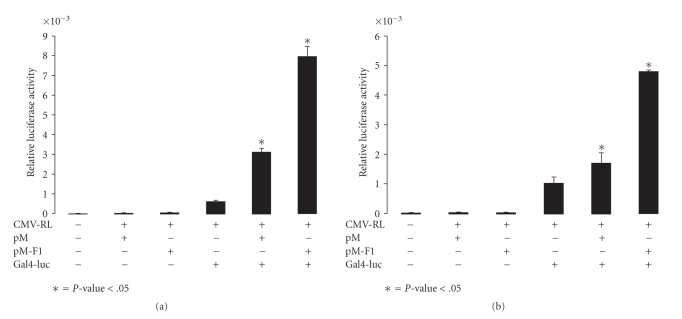
PRIC295 functions as a coactivator protein in Gal4-binding assay. Transfection of chimeric Gal4-DBD-PRIC295-F1(E) or Gal4-DBD-PRIC295-F2(F) significantly enhanced transcription of the luciferase reporter gene. Transfection of chimeric Gal4-DBD-PRIC295-F3 did not significantly enhance the transcription of the luciferase reporter (F3; not illustrated). HeLa cells were transfected with 100 ng of pM-ΔPRIC295^1–915^ (F1) (a), pM-ΔPRIC295^840–1815^ (F2) (b), Gal4-TK-Luc and 50 ng of CMV-RL as indicated (+ or −). Each column shown is the mean relative luciferase unit value for triplicate experiments. Statistical significance was calculated by comparison of transfected groups marked with an *.

To further confirm the transactivational activity of PRIC295, a Gal4-binding assay was performed using the yeast Gal4-DBD fused to the PRIC295 fragments, ΔPRIC295^1–915^ (F1), ΔPRIC295^840–1815^ (F2), or ΔPRIC295^1740–2671^(F3). These chimeric proteins also showed the ability to significantly enhance transcription of the luciferase reporter under the direction of the thymidine kinase promoter fused to the c-terminal of Gal4. Cells transfected with the chimeric PRIC295-F1 fragment showed a statistically significant (*P*-value = .000748 for Gal4-DBD-F1 and  .001733 for Gal4-DBD-F2) increase in relative luciferase activity as compared to cells transfected with only the Gal4-DBD (Figure 6). Gal4-DBD-F3 exhibited only a modest increase in the activity (not illustrated). These data clearly demonstrate the activity of PRIC295 as a coactivator protein though the exact mechanism by which PRIC295 exerts this influence is presently unclear.

### 3.6. PRIC295 Binding Partners

**Figure 7:**
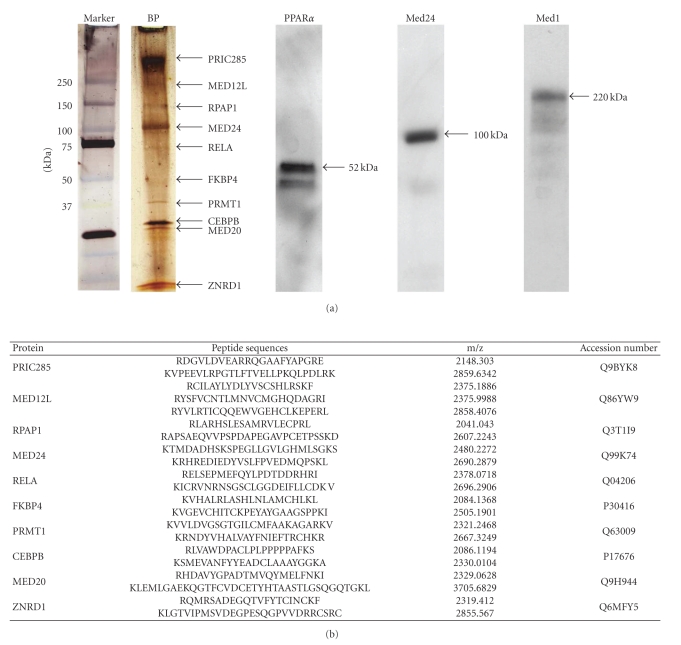
PRIC295-interacting proteins. (a) PRIC295-interacting proteins were isolated using GST-ΔPRIC295^840–1815^ (F2) (pGEX-ΔPRIC295^840–1815^) to pull down interacting proteins from rat liver nuclear extract. Selected bands (lane 2-BP; arrows) were excised from the 4–20% Tris-HCl gel and digested with trypsin to release peptide fragments. (b) Peptide fragments derived from excised bands were subjected to MALDI-TOF analysis. Proteins matching the identities with peptide fragments are shown with some matching peptide sequences, m/z ratios, and accession numbers. In (a), immunoblotting of ciprofibrate-binding protein complex reveals the presence of PPAR*α* (lane 3), Med24/TRAP100 (lane 4), and Med1 (lane 5).

We have previously demonstrated that coactivator-binding protein PIMT (NCoA6IP) interacts with PRIP (ASC-2/NCoA6), CBP, p300, and Med1 to presumably form a PIMT complex [62]. PRIP/ASC-2 also interacts with other cofactors to form a steady complex described as ASCOM (for ASC-2 complex) [68]. ASCOM contains histone H3-lysine-4 (H3LK4) methyltransferase MLL3 or its paralogue MLL4 [68]. Interaction between ASCOM and the ATPase-dependent chromatin remodeling complex Swi/Snf have also been demonstrated and these interactions promote the binding of these complexes to nuclear receptor target genes [69]. Since coactivator and coactivator-associated protein interactions are being recognized with increasing frequency, we decided to investigate whether PRIC295 associates with other cofactors. For this purpose, we chose to use ΔPRIC295^840–1815^ (F2) region fused to GST at the N-terminus of the protein to affinity pulldown potential PRIC295 binding partners from rat liver nuclear extracts (Figure 7(a)). PRIC295-F2 was chosen because this fragment with 4 LXXLLs generally bound to all nuclear receptors analyzed in this study, and also we could not successfully express full length PRIC295 as GST-fusion protein. MALDI-TOF mass spectrographic analysis of selected PRIC295 F2 fragment bound peptides (Figure 7(a)) resolved by SDS-PAGE revealed the presence of PRIC285 [Q9BYK8] [33], Med12L [Q86YW9], Med24 [Q99K74] [60], PRMT1 [Q63009] [70], C/EBP*β* [P17676] [71], Med20 [Q9H944] [72], and ZNRD1 [Q6MFY5] [73] among others (Figure 7(b)). To confirm some of these potential interactions, we performed immunoblot analysis for the presence of PPAR*α*, Med24, and Med1 in the ciprofibrate bound protein complex (Figure 7(a)). These immunoblots established the presence of Med24 and Med1 among the proteins pulled down using ciprofibrate-Sepharose slurry that appear to form PRIC295 complex (PRIC295COM).

### 3.7. PRIC295 Localizes to the Nucleus and Interaction between PRIC295 and PPAR, Med24, and Med1 in Intact Cells

**Figure 8:**
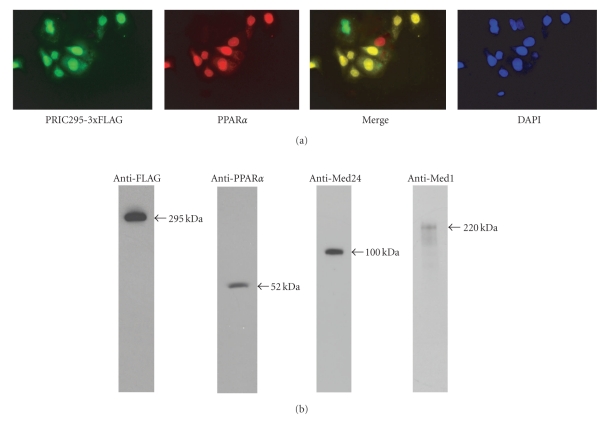
Intracellular localization of PRIC295. (a) Immunofluorescence microscopy to visualize intracellular localization of PRIC295-3xFLAG and PPAR*α* in HeLa cancer cells after transfection with pcDNA-PRIC295-3xFLAG and pCMX-PPAR*α*. Anti-FLAG M2 monoclonal antibody was used for PRIC295-3xFLAG (green color), and anti-PPAR*α* antibody was used to stain PPAR*α* (red color). DAPI nuclear stain is for the visualization of cells in the field. Merged image of PRIC295-3xFLAG (green) and PPAR*α* (red) reveals yellow coloration of nuclei that coexpress both proteins. The presence of a green and red cell in the merged panel is an indication that these cells are not likely transfected with both plasmids. (b) Immunoprecipitation data showing purified PRIC295-3xFLAG protein and coimmunoprecipitated proteins PPAR*α*, Med24/TRAP100, and Med1. Cells were transfected with pcDNA-PRIC295-3xFLAG only.

To confirm the subcellular localization of PRIC295, pcDNA-PRIC295-3xFLAG was transfected into HeLa cells. Immunofluorescence microscopy revealed the nuclear localization of FLAG-tagged PRIC295 (Figure 8(a); panel PRIC295-3xFLAG). We also cotransfected these cells with pCMX-PPAR*α* and using anti-PPAR*α* antibodies we confirmed the expression of this receptor in the nucleus (Figure 8(a); panel PPAR*α*). When PRIC295 and PPAR*α* images were merged most nuclei were colored yellow indicating a high degree of overlap in these cells that express both proteins (Figure 8(a); merged image) but further high resolution studies are needed to determine if these two proteins are colocalized in the nucleus. Analysis of several cells indicated that some cells were expressing PRIC295 while others only PPAR*α* (Figure 8(a); one red and one green nucleus in addition to several yellow colored nuclei). DAPI was used to stain all nuclei (Figure 8(a), DAPI). 

We used immunoprecipitation followed by immunoblotting to establish the *in vivo* interaction of PRIC295 with PPAR*α*, Med24, and Med1 (Figure 8(b)). For this purpose, we transfected cells with PRIC295-3xFLAG and immunoprecipitated with FLAG antibodies and immunoblotted the precipitates with antibodies against PPAR*α*, Med24, and Med1 (Figure 8(b)). Clearly, this approach established the presence of coimmunoprecipitated PPAR*α*, Med24, and Med1 (Figure 8(b)). These observations demonstrate that PRIC295 interacts *in vivo* not only with PPAR*α* but also with two members of the Mediator complex in living cells. Further studies will be necessary to analyze nuclear proteins that bind to all three PRIC295 fragments, F1, F2, and F3, to fully delineate the PRIC295COM.

### 3.8. GST-Pulldown to Establish PRIC295 Interaction with Med1 and Med 24 In Vitro

**Figure 9:**
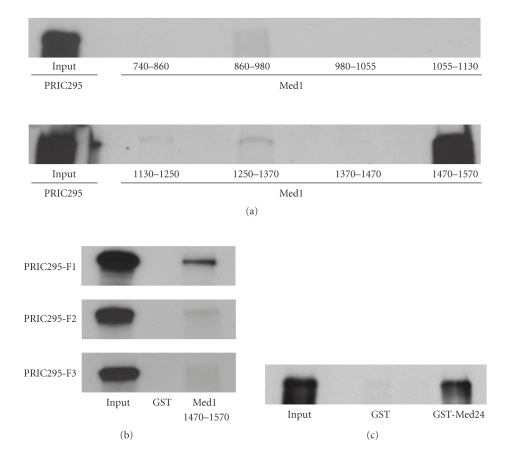
PRIC295 interacts with Med1. (a) *In vitro* translated and radiolabeled PRIC295 protein interacts with the known PPAR*α* coactivator, Med1, as assessed by GST-pulldown approach using GST-fusion protein fragments of the PBP protein. Interaction between PRIC295 and GST-ΔPBP6^1470–1570^ appears robust. (b) GST-pulldown of radiolabeled ΔPRIC295^1–915^ (F1), ΔPRIC295^840–1815^ (F2), and ΔPRIC295^1740–2671^ (F3), respectively, using GST-ΔPBP^1470–1570^ showing a strong binding interaction occurring between ΔPRIC295^1–915^ (F1) and GST-ΔPBP^1470–1570^.

Further experimentation was performed to confirm the interaction of PRIC295 with Mediator complex proteins Med1 and Med24 *in vitro *(Figures 9(a)–9(c)). *In vitro* translated and radiolabeled full-length PRIC295 was allowed to interact with GST-Med1 fragments (Figure 9(a)). Fragments covering Med1 aa 1–740 (not shown), 740–860, 860–980, 980–1055, 1055–1130, 1130–1250, 1250–1370, and 1370–1470 failed to bind with full-length PRIC295 (Figure 9(a)). However, PRIC295 bound strongly with a fragment from the C-terminal of Med1 containing amino acids 1470–1570 (Figure 9(a)). Coomassie-stained GST-Med1 fusion proteins used in these assays are shown in Supplementary Figure 5. Analysis of this region of Med1 showed a lack of any known conserved domains or binding sites. This Med1 fragment is devoid of any LXXLL motif. In order to ascertain the region of the PRIC295 that binds to Med1 aa 1470–1570, GST-pulldowns were performed using *in vitro* translated PRIC295 fragments F1, F2, and F3 and found that F1 interacts strongly with Med1 but the other PRIC295 fragments revealed minimal binding (Figure 9(b)). Finally, GST-pulldowns also showed that PRIC295 interacts with Med24 (Figure 9(c)). 

During the past 15 years, several proteins have been identified which interact with PPARs and other nuclear receptors and serve as transcription cofactors/coregulators [18, 55, 74]. We have been using the MALDI-TOF approach to identify and characterize PPAR*α* or its ligand binding proteins referred to as PRIC complex [33, 34]. Using this mass spectrography-based technique, we now report the identification of a novel coactivator, PRIC295. PRIC295 revealed a strong, ligand-dependent interaction not only with PPAR*α*, but with several other nuclear receptor proteins. Of considerable interest is that this protein contains several LXXLL and HEAT repeat motifs that appear to be important in protein-protein interactions and in transcription [67]. This may indicate that PRIC295 has a role in mediating the transcription of target genes for several members of the nuclear receptor superfamily, though further investigation of this interaction and its role with those receptor proteins *in vivo* is warranted.

This study also utilized MALDI-TOF procedures to delineate PRIC295 binding protein complex (PRIC295COM). Analysis revealed PRIC295 binding proteins that included PRIC285, Med12L, Med20, and Med24 suggesting that PRIC295 plays a potentially important role in transcription. For example, it should be noted that PRIC285 contains a UvrD helicase motif and has been previously shown to enhance transcriptional activation mediated by PPAR*α* [33]. The association of PRIC295 with Med12L, a putative homologue of Med12 subunit of Mediator complex, and with Med1, Med20, and Med24, which are members of the Mediator complex suggests that PRIC295 may be involved as a platform protein in the formation of the large transcription complex [33, 34, 75, 76]. While Med1 has been studied extensively (see [77]; Viswakarma et al., PPAR Research in press) less is known about Med24 and Med20 [77]. Med24 was initially identified as a cofactor important for mediating transcription by thyroid hormone and vitamin D receptors which interacts with and localizes together with Med1/TRAP220 [60]. Med24 may also have additional roles in gene regulation outside of its role as a member of the Mediator complex [78]. Med20 was first identified as a human homologue of the *Drosophila *TRF-proximal protein (hTRFP) and was shown to be able to enhance the transcriptional activity of RNA Pol II [72]. Med20, together with Mediator complex subunits Med8 and Med18 plays an important role in the proper folding and formation of one of the subunits of the Mediator complex [79]. PRMT1 is an arginine methyltransferase which is identified in the complex of PRIC295-interacting proteins. PRMT1 was identified by its interaction with the TIS21 and BTG1 proteins and has been shown to have important roles in RNA processing and transcription [70, 80]. PRMT1 potentiates the activity of PGC-1*α*, a known coactivator of PPARs [81]. The role of PRMT1 in RNA processing and transcription has been shown to be important part in mediating viral infection [82]. C/EBP*β* is a transcription factor that is known to be involved in processes mediated by PPARs including adipogenesis and the induction of endoplasmic reticulum stress [71, 83, 84]. In this study, we have identified C/EBP*β* in the complex of proteins interacting with PRIC295. Zinc ribbon domain-containing 1 (ZNRD1) was also among the complex of PRIC295-interacting proteins. ZNRD1 has a role in regulating ERCC1 and Bcl-2 which are important in cancer progression though the mechanism by which it does this is presently unclear [85]. ZNRD1 also appears to have an influence in the progression of HIV infection [86]. 

Evidence suggests that certain coactivators are essential for the effective transcriptional activation of PPAR*α* target genes [57, 87]. Conditional deletion in the mouse liver of the coactivator MED1 results in impaired liver regeneration following partial hepatectomy [57]. Absence of Med1 results in the abrogation of PPAR*α* ligand induced pleiotropic responses including hepatocarcinogenesis [57, 87]. Deficiency of Med1 also prevents acetaminophen-induced hepatotoxicity [87]. On the other hand, absence of SRC-1, SRC-2, and SRC-3 had no effect on PPAR*α* signaling (see [88]; Viswakarma et al., PPAR Research in press). Med1 is essential for the interaction of the activated PPAR*α*/RXR*α* heterodimer with RNA polymerase II and the basal transcription machinery of the cell [89]. In addition, germ-line deletion of Med1 results in embryonic lethality illustrating the important roles that coactivator proteins may be playing in other tissues [90–92]. Given this information, the interaction of PRIC295 with Med1, Med24, and some other members of the Mediator complex of proteins is of particular interest.

## 4. Conclusions

In the recent past, many new coactivator proteins that are involved in the transcriptional regulation of the expression of PPAR*α* target genes have been discovered and studied. These coactivators grant a way to regulate the transcriptional ability of PPAR*α* and other nuclear receptors in a tissue and cell-specific manner. Our studies show that PRIC295 is a nuclear receptor coactivator which interacts with PPAR*α* and several other nuclear receptors and may play a larger role in the transcription of target genes through nuclear receptors. The association of proteins such as Med1, Med24, and Med20, the members of Mediator complex with PRIC295 suggests the existence *in vivo* of a complex of proteins designated PRIC295COM.

## Supplementary Material

Supplementary Material includes a Supplementary Table and 5 Supplementary Figures. The Supplementary Table contains a list of the amino acid locations of HEAT repeats within the PRIC295 protein sequence. The Supplementary Figures contain quatitative PCR data of whole mouse embryo expression of PRIC295 at several timepoints (Supplementary Figure 1), GSTpulldowns of full-length PRIC295 with several nuclear receptors at reduced ligand concentrations (Supplementary Figure 2), a quantitative measurement of the binding between PRIC295 fragments and several nuclear receptors (Supplementary Figure 3), transactivation data for RXR*α* using cognate ligand (Supplementary Figure 4) and coommassie-stained loading controls for GST-pulldowns performed using Med1 fragments (Supplementary Figure 5).
