# Supplementary material for: PRIC295, a Nuclear Receptor Coactivator, Identified from PPARα-Interacting Cofactor Complex
Source: PPAR Res. 2010 Sep 5;2010:173907. doi: 10.1155/2010/173907 (PMC2946606; doi:10.1155/2010/173907)
Supplement: Supplementary file 1 — Supplementary Material includes a Supplementary Table and 5 Supplementary Figures. The Supplementary Table contains a list of the amino acid locations of HEAT repeats within the PRIC295 protein sequence. The Supplementary Figures contain quatitative PCR data of whole mouse embryo expression of PRIC295 at several timepoints (Supplementary Figure 1), GSTpulldowns of full-length PRIC295 with several nuclear receptors at reduced ligand concentrations (Supplementary Figure 2), a quantitative measurement of the binding between PRIC295 fragments and several nuclear receptors (Supplementary Figure 3), transactivation data for RXRα using cognate ligand (Supplementary Figure 4) and coommassie-stained loading controls for GST-pulldowns performed using Med1 fragments (Supplementary Figure 5). [file 173907.f1.pdf]

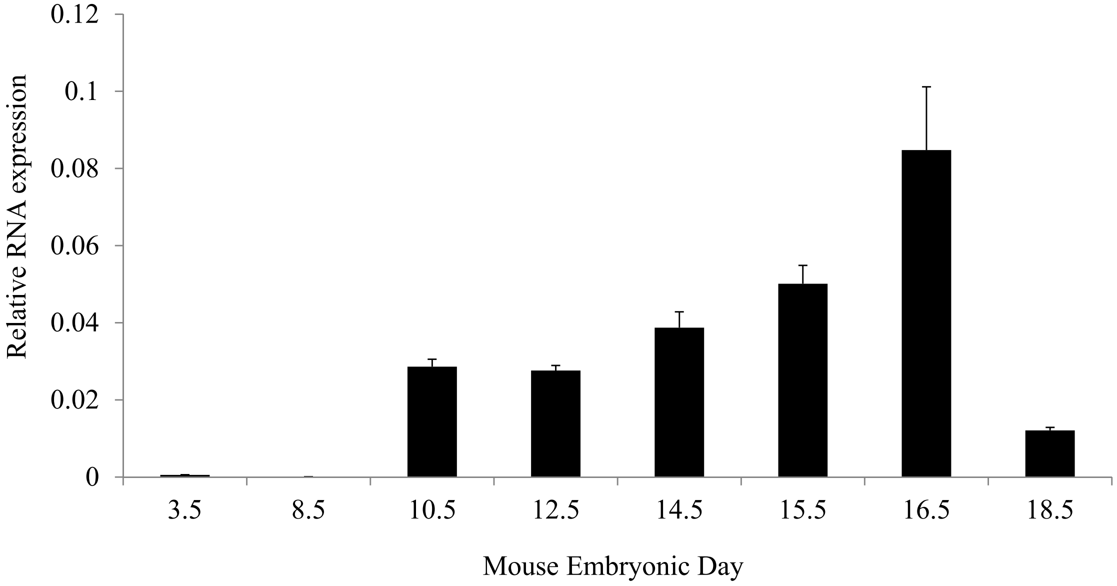

SUPPLEMENTARY FIGURE 1: Quantitative PCR data showing the mean expression of PRIC295 RNA during mouse embryonic development at several time points. RNA from 3 whole embryos per time point were prepared and pooled. Samples were run in triplicate to give the measured mean values with standard deviations shown.
