# Supplementary material for: PRIC295, a Nuclear Receptor Coactivator, Identified from PPARα-Interacting Cofactor Complex
Source: PPAR Res. 2010 Sep 5;2010:173907. doi: 10.1155/2010/173907 (PMC2946606; doi:10.1155/2010/173907)
Supplement: Supplementary file 2 [file 173907.f2.pdf]

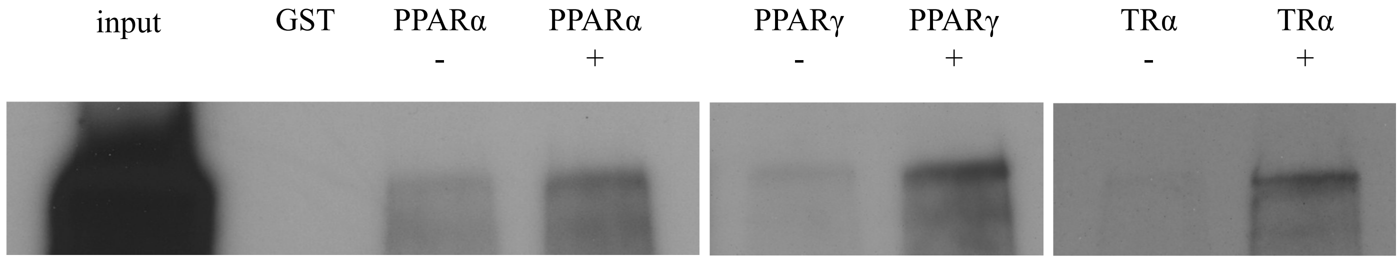

SUPPLEMENTARY FIGURE 2: GST-pulldowns conducted using *in vitro*-translated full-length PRIC295 and selected GST-fusion receptor proteins PPARα, PPARγ, and TRα at a ligand concentration of 10 μM. Each receptor shows increased binding interaction with PRIC295 in the presence of ligand. Ligands used were Wy-14,643 for PPARα, rosiglitizone for PPARγ, and triiodothyronine for TRα. Pulldowns were done in the absence (-) or presence (+) of ligand as indicated.
