# Supplementary material for: PRIC295, a Nuclear Receptor Coactivator, Identified from PPARα-Interacting Cofactor Complex
Source: PPAR Res. 2010 Sep 5;2010:173907. doi: 10.1155/2010/173907 (PMC2946606; doi:10.1155/2010/173907)
Supplement: Supplementary file 3 [file 173907.f3.pdf]

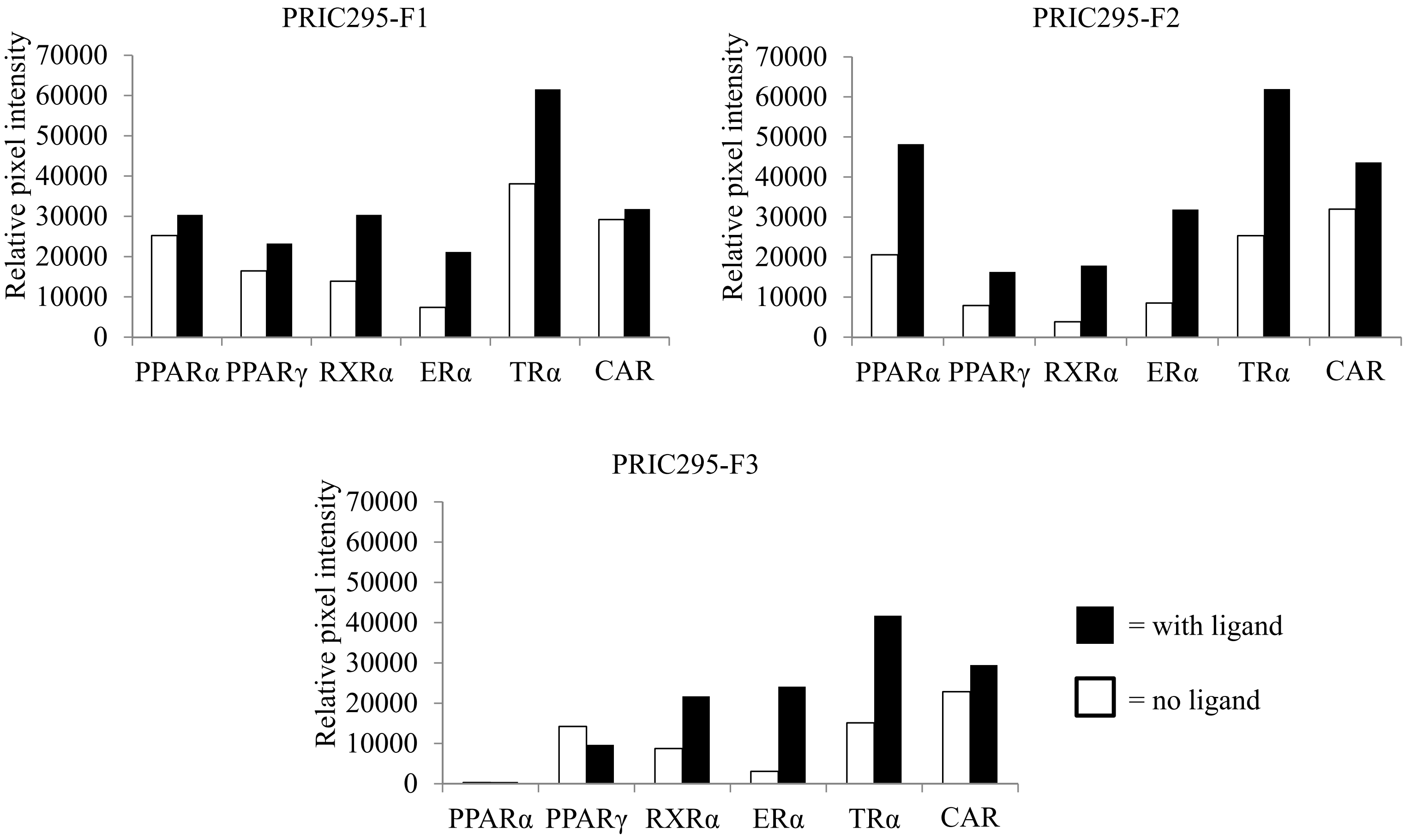

SUPPLEMENTARY FIGURE3: Digital image quantification of in vitro binding interaction between radiolabeled PRIC295-F1 (ΔPRIC295$^{1\text{-}915}$), PRIC295-F2 (ΔPRIC295$^{840\text{-}1815}$) and PRIC295-F3(ΔPRIC295$^{840\text{-}1815}$) with GST-fusion nuclear receptor proteins as indicated. Open bars indicate interaction in the absence of ligand. Dark bars indicate interaction in the presence of receptor-specific ligand. Individual ligands used are as previously described
