# Supplementary material for: PRIC295, a Nuclear Receptor Coactivator, Identified from PPARα-Interacting Cofactor Complex
Source: PPAR Res. 2010 Sep 5;2010:173907. doi: 10.1155/2010/173907 (PMC2946606; doi:10.1155/2010/173907)
Supplement: Supplementary file 4 [file 173907.f4.pdf]

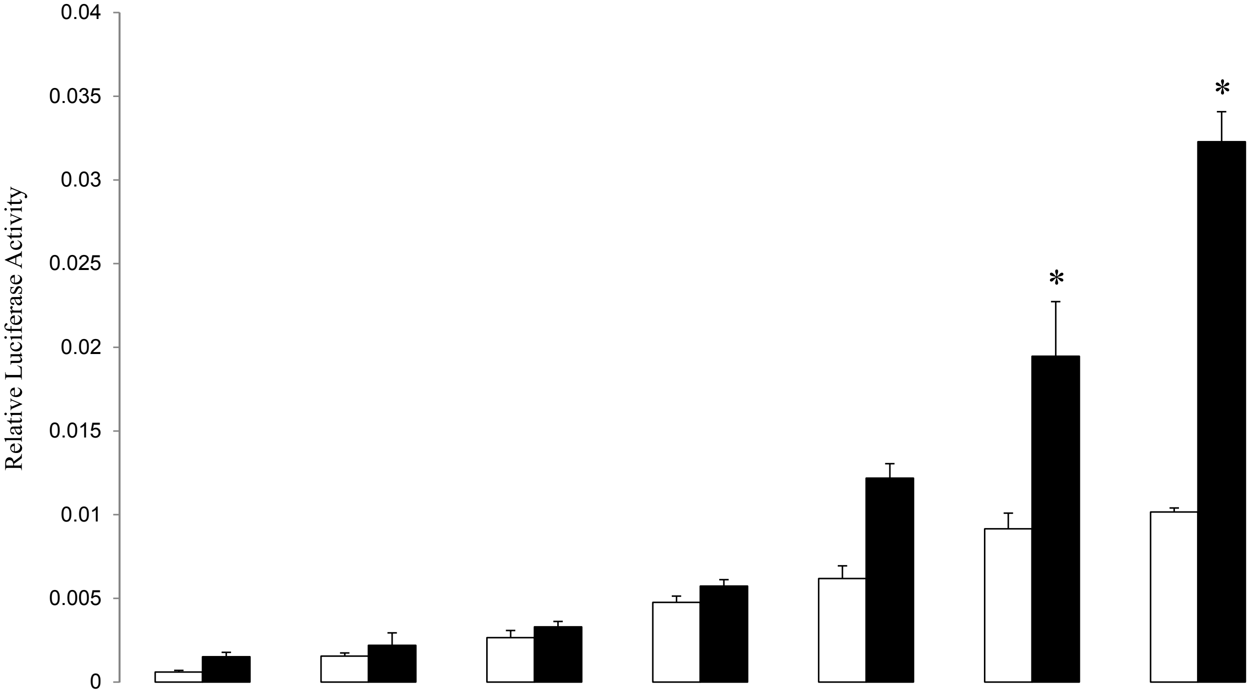

CMV-RL	-	+	+	+	+	+	+
pGL3	-	+	-	-	-	-	-
3xPPRE	-	+	+	+	+	+	+
PPARα	-	-	-	-	+	+	+
RXRα	-	-	-	+	-	+	+
PRIC295	-	-	+	-	-	-	+

□ = no ligand ■ = with ligand * = p-value < .05

SUPPLEMENTARY FIGURE 4: PRIC295 functions as a transcriptional coactivator for heterodimer partner RXRα. HeLa cells were transfected with 100 ng each of pcDNA-PRIC295, pCMX-PPARα, pCMX-RXRα and pGL3-3xPPRE and 50 ng of CMV-RL as indicated (- or +). Each column represents the mean relative luciferase unit value of experiments conducted in triplicate. Open bars represent activity in the absence of ligand and dark bars represent activity in the presence of the RXRα ligand, 9-cis-retinoic acid. Values were normalized against the expression of *Renilla* under the control of the CMV promoter. Statistical significance was tested using a one-tailed student's t-test by comparing transfected groups marked with an *.
