# Supplementary material for: PRIC295, a Nuclear Receptor Coactivator, Identified from PPARα-Interacting Cofactor Complex
Source: PPAR Res. 2010 Sep 5;2010:173907. doi: 10.1155/2010/173907 (PMC2946606; doi:10.1155/2010/173907)
Supplement: Supplementary file 5 [file 173907.f5.pdf]

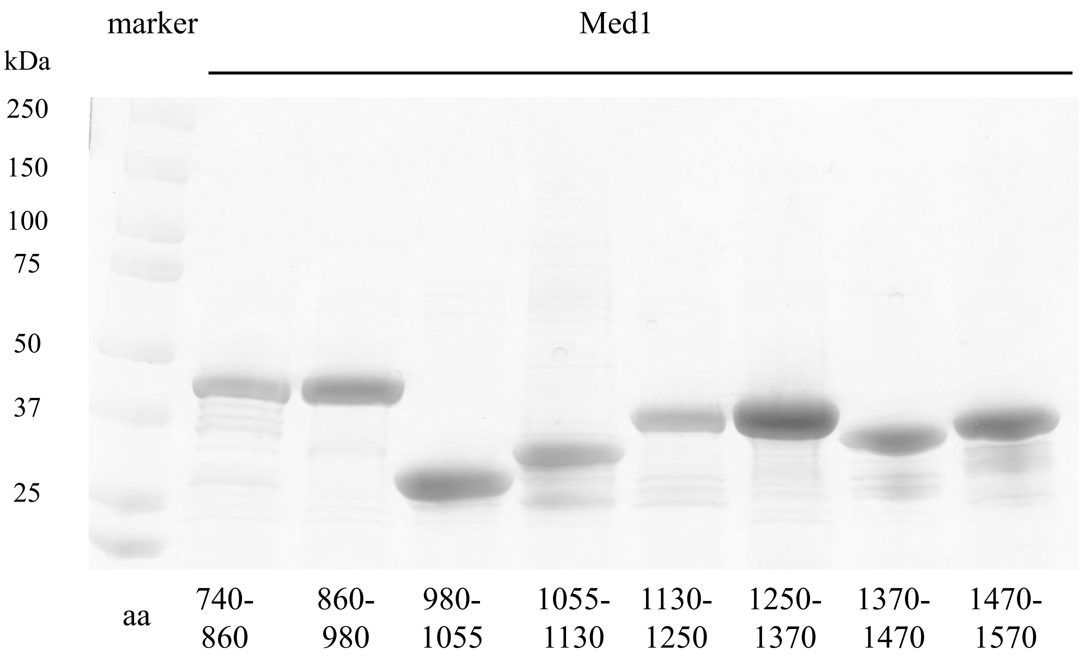

SUPPLEMENTARY FIGURE 5: Coomassie-stained GST-fusion protein fragments of Med1 used in Fig. 8A. Lanes contain, from left to right, marker, GST-Med1 740-860, 860-980, 980-1055, 1055-1130, 1130-1250, 1250-1370, 1370-1470, and 1470-1570.
