# Supplementary material for: PRIC295, a Nuclear Receptor Coactivator, Identified from PPARα-Interacting Cofactor Complex
Source: PPAR Res. 2010 Sep 5;2010:173907. doi: 10.1155/2010/173907 (PMC2946606; doi:10.1155/2010/173907)
Supplement: Supplementary file 6 [file 173907.f6.pdf]

SUPPLEMENTARY TABLE 1: A list of HEAT repeat locations within the PRIC295 protein according to their initial and ending amino acid residues.

Repeat	Location (aa)
H_1	257-294
H_2	295-331
H_3	460-503
H_4	1078-1115
H_5	1290-1332
H_6	1335-1372
H_7	1455-1492
H_8	1493-1530
H_9	1534-1571
H_{10}	1573-1609
H_{11}	1611-1648
H_{12}	1653-1690
H_{13}	1773-1810
H_{14}	1812-1848
H_{15}	1921-1958
H_{16}	1959-1996
H_{17}	2001-2038
H_{18}	2039-2076
H_{19}	2188-2225
H_{20}	2259-2296
H_{21}	2339-2380
H_{22}	2422-2459
H_{23}	2560-2583
H_{24}	2588-2625
